# How Coping Combination Affects Innovation Ambidexterity in Business Failure Situations

**DOI:** 10.3389/fpsyg.2020.01409

**Published:** 2020-07-14

**Authors:** Jinliang Chen, Feng Jiang, Song Lin

**Affiliations:** School of Business, Central University of Finance and Economics, Beijing, China

**Keywords:** coping combination, loss orientation coping, restoration orientation coping, innovation ambidexterity, business failure, entrepreneurial cognition

## Abstract

As an effective cognitive and behavioral strategy, coping helps to overcome negative events. Although coping and its effects have been widely studied in psychology, little is known about the combination of entrepreneurs’ coping and its connection with firms’ innovation ambidexterity. To fill these gaps, in this study, the authors collected 106 samples through two serial-wave surveys of the Bohai Economic Rim in China and tested the theoretical hypotheses using polynomial regression with response surface analysis. The results showed that alignment coping combination enhanced innovation ambidexterity by reshaping an entrepreneur’s cognitive structure. Misalignment coping combination was found to enhance innovation ambidexterity by eliciting an entrepreneur’s different types of information processing systems. This study contributes to the literatures of coping, innovation ambidexterity, and upper echelons theory from the entrepreneurial cognition approach.

## Introduction

Just as a coin has two sides, business failure is simultaneously associated with negative and positive effects (McGrath, 1999; Ucbasaran et al., 2013). Scholars have extensively studied the use of coping in mitigating the negative consequences of business failure that could overpower and jeopardize the positive aspects (Shepherd, 2003; Singh et al., 2007; Shepherd et al., 2011). The extant studies on coping (Shepherd, 2003; Folkman and Moskowitz, 2004; Shepherd et al., 2011; Biggs et al., 2017; Shepherd and Patzelt, 2018) have revealed valuable insights. To review the current scholarly investigations, two research gaps remained. The first gap is that the current scholarly knowledge mainly centers on coping at the individual level while relatively ignoring the organizational level. Following the Shepherd (2003), for example, an increasing number of studies investigate entrepreneurs’ individual recovery from the grief triggered by business failures (Shepherd and Patzelt, 2018). In contrast, there is still a dearth of research that systematically outlines the effect of entrepreneurs’ coping, although the thoughts of coping’s effect at the organizational level was highlighted in literature (Carter et al., 1996; Shelton, 2006).

The second gap is that the current scholarly investigations account for the paucity of the more precise coping combinations. Previous coping studies were both grounded in the traditional classification of problem-focused and emotion-focused coping (Folkman and Moskowitz, 2004) and referenced the dual-process model of coping bereavement (Stroebe and Schut, 1999). In so doing, the previous investigations studied the concept of oscillation orientation coping based on loss orientation coping (LOC) and restoration orientation coping (ROC) (Shepherd, 2003; Shepherd et al., 2011). Although the previous scholarly investigations accorded thoughtful insights of coping combination to this study, oscillation orientation coping was merely an uneven coping combination that interchanges between LOC and ROC, and the more precise coping combinations between LOC and ROC were not really known (Biggs et al., 2017; Shepherd and Patzelt, 2018). The coping combination should therefore be more thoroughly investigated.

The upper echelons theory (Hambrick and Mason, 1984; Hambrick, 2007) holds that an entrepreneur’s cognition in strategic decision-making inevitably influences firm-level outcomes (Bromiley and Rau, 2016). More specifically, studies on the underlying logic of entrepreneurial cognition (Mitchell et al., 2002; Randolph-Seng et al., 2015) revealed that coping reshaped an entrepreneur’s cognitive structure and elicited different types of information processing systems (Burns and D’Zurilla, 1999; Ucbasaran et al., 2009), that influenced firm-level outcomes by exerting an impact on an entrepreneur’s strategic decision-making. Therefore, the importance of studying the effect of coping at the firm level is highlighted on the basis of the entrepreneurial cognition approach, although the initial purpose of coping is to overcome stress at an individual level.

Furthermore, opportunity creation (Alvarez and Barney, 2007) is a core feature of entrepreneurship, and product innovation is significantly correlated with the potential of opportunity creation (Ramoglou and Tsang, 2016, 2017). According to Jansen et al. (2006), two types of product innovation have attracted a significant amount of scholarly attentions: exploitative and exploratory innovation. Innovation ambidexterity indicates a firm’s ability to pursue exploitative and exploratory innovation, and ambidextrous innovation refers to the activities a firm to develop the two types of innovation. These two academic-valuable concepts have been extensively explored in the existing literature (Wang et al., 2019). Following Wang et al. (2019), innovation ambidexterity is characterized as a form of dynamic capability, which makes it difficult for competitors to imitate. Hence, it helps firms to perform innovation activities with effectiveness and efficiency, consequently contributing to firms’ competitive advantage. Keeping those in mind, this study investigated innovation ambidexterity as the firm-level outcome, with the above impact of innovation ambidexterity.

Considering the abovementioned aspects, this study aimed to bridge the two stated gaps in extant researches by focusing on coping combinations by visiting the functioning of coping combinations and vis-à-vis innovation ambidexterity. Alignment coping combination (ACC) is defined as the alignment shifting between LOC and ROC with a balanced relationship indicating that LOC is equal to ROC. Misalignment coping combination (MCC) is defined as the misalignment shifting between LOC and ROC, indicating that either LOC is larger than ROC or ROC is greater than LOC. Both ACC and MCC in this study were designed by using response surface analysis technology. Thereafter, the alignment and misalignment combination between LOC and ROC, and their associations with innovation ambidexterity (firm-level outcome), were respectively tested through response surface analysis technology. Finally, the “bowl” relationship image was graphed in a three-dimensional space to provide a geometric intuition of the complex relationships (Edwards, 2002; Cafri et al., 2010).

These arguments were tested with data collected through two serial-wave surveys conducted in China’s Bohai Economic Rim (BER). Using polynomial regression with response surface analysis, a U-shape relationship was found between ACC and innovation ambidexterity. The relationship was positive when ACC was relatively high, and it was negative when ACC was relatively low. It was also determined that MCC was positively related to innovation ambidexterity. Specifically, innovation ambidexterity grew either when LOC increased and ROC decreased, or when ROC increased and LOC decreased.

These findings suggest three possible theoretical contributions. First, this study contributes to the upper echelons theory through the entrepreneurial cognition approach that allows it to focus on the functioning of ACC and MCC on innovation ambidexterity. Thus, the general underlying logic of the psychological characteristics mentioned in the upper echelons theory research stream is extended. Second, this study adds to the underlying theoretical logic of coping mechanisms that work at the organizational level. Grounded in the entrepreneurial cognition approach, the results of this study disclose the effects of coping combinations in strategic decision-making, by revealing the relationships between entrepreneurs’ coping combinations and innovation ambidexterity. Third, this study introduces the effects of coping on business failure from the information processing perspective. Specifically, a more precise underlying mechanism that reshapes an entrepreneur’s cognitive structure was intensively uncovered based on ACC. A more precise underlying mechanism that elicited an entrepreneur’s different types of information processing systems was also revealed through the exploration of the effects of MCC.

The next section overviews the relevant theories and hypotheses pertaining to the effects of ACC and MCC. A subsequent section describes the data collection, scale, and analytical techniques utilized for the present investigation. Thereafter, the paper presents the empirical results obtained through polynomial regression with response surface analysis. Finally, the results, theoretical contributions, and managerial implications are discussed along with the acknowledgment of certain limitations and directions for future research.

## Theory and Hypotheses

### Coping With Business Failure

The issue of failure is a longstanding debate in the scholarly entrepreneurship literatures. The causes and consequences of business failure are the two dominant streams (Shepherd, 2013; Khelil, 2016). As studies addressing the causes have increased (Artinger and Powell, 2015; Tingbani et al., 2019), numerous research initiatives have probed the consequences (Singh et al., 2015; Amankwah-Amoah and Wang, 2019). Studies on the consequences have found simultaneously negative and positive outcomes (McGrath, 1999; Ucbasaran et al., 2013). On the one hand, business failure causes a personal loss for entrepreneurs in the form of financial debt (Cope, 2011), breakdown in marriage (Singh et al., 2007), and stigma (Sutton and Callahan, 1987), all of which trigger negative emotions (Shepherd, 2009). On the other hand, entrepreneurs benefit from business failures by acquiring “general knowledge” from their failures and initiating sense-making process to move forward (Minniti and Bygrave, 2001; Shepherd, 2003, 2009).

As ways to govern the entrepreneurial learning process and mitigate the negative effects of business failure, a regulation approach that utilizes coping strategies and a normalization approach that depends on standardized processes has been discussed (Shepherd et al., 2009; Shepherd and Patzelt, 2018). However, the normalization approach imposes certain limitations such as diminishing learning benefits, and reducing subsequent commitments and, thus, studies have not extensively explored this perspective. Conversely, as a core concept of the regulation approach, coping has been observed to increase realistic thinking and to resolve stress (Lazarus and Folkman, 1984; Singh et al., 2007). The regulation approach has thus attracted considerable scholarly attention (Cope, 2011; Ucbasaran et al., 2013). Three categories have been widely studied in the coping research: LOC, ROC, and the combination of both labeled oscillation orientation coping (Shepherd, 2003; Shepherd et al., 2011). In addition, two classifications, problem-focused and emotion-focused coping, have been comprehensively investigated for their functioning in overcoming the negative effects of business failure (Singh et al., 2007).

Coping, to regulate the stressor itself or to manage emotion, was advanced when the transactional theory of emotion and coping was postulated (Folkman and Moskowitz, 2004). According to the transactional theory, the regulation of the stressor itself was named problem-focused coping, and emotion-focused coping indicated the management of emotion (Lazarus and Folkman, 1984). This dichotomy offered a viable means with which to explore the different kinds of coping strategies, thus scholarly literatures on the topic increased as a result (Biggs et al., 2017). However, the development of the studies was accompanied by critiques pertaining to the need for conceptually clear, mutually exclusive, and exhaustive or comprehensive taxonomies (Biggs et al., 2017). Accordingly, researchers proposed alternative taxonomies such as the five coping types categorized on the basis of cybernetic theory (Edwards, 1992), or the three types of coping postulated according to adaptive functions (Skinner et al., 2003). Further, coping combination was also explored, as typified by oscillation orientation coping based on LOC and ROC (Stroebe and Schut, 1999; Shepherd, 2003; Shepherd et al., 2011). The present study followed the coping combination research to delve into the coping combination using the response surface analysis technology.

In psychology, coping was originally used to protect the mental and physical health of individuals from harmful stressors through individual thoughts and behaviors with regard to personal associations with the individual’s environment (Folkman and Moskowitz, 2004; Biggs et al., 2017). In entrepreneurship also, coping was believed to work at an individual level in helping an entrepreneur recover from the negative emotions triggered by business failure while also retaining the entrepreneur’s learning from the experience of failure (Shepherd, 2003; Shepherd and Patzelt, 2018). Although the extant literatures on coping have elucidated the recovery process from negative emotion in tandem with entrepreneurial learning (Shepherd et al., 2011; Shepherd and Patzelt, 2018), knowledge on its benefit at the firm level is still lacking.

### Entrepreneurship and Innovation Ambidexterity

Opportunity is thought to be the most critical element of entrepreneurship according to the fundamental theoretical perspective (Kirzner, 1979; Shane and Venkataraman, 2000). From this starting point, a growing number of entrepreneurship studies have explored the question: “Where does opportunity originate?” (Suddaby et al., 2015). This argument is known as the origin of entrepreneurial opportunity, and it became the core conundrum of the purview of entrepreneurship. The two dominant views in this debate are the discovery and the creation approach (Alvarez and Barney, 2007). The basic underlying opinion of the discovery approach is that entrepreneurial opportunity exists objectively. The creation approach, on the other hand, believes that entrepreneurial opportunity is subjective, indicating that entrepreneurial opportunity is endogenously created.

An ontological analysis was undertaken by Ramoglou and Tsang (2016) to uncover this core conundrum. Later, the discourse continued for several years in the *Academy of Management Journal* (Alvarez et al., 2017; Berglund and Korsgaard, 2017; Ramoglou and Tsang, 2017, 2018; Danneels and Braver, 2018). The relationship between innovation and opportunity creation became pivotal as the core conundrum debate progressed. According to Ramoglou and Tsang (2016, p. 411), entrepreneurial opportunity was defined as “the propensity of market demand to be actualized into profits through the introduction of novel products or services.” Generally, scholars agreed that product innovation is significantly correlated to the potential of creating opportunities even though the synonymity of product creation and opportunity creation was believed to be erroneous (Ramoglou and Tsang, 2016, 2017; Danneels and Braver, 2018; Wu et al., 2020).

Keeping the relationship between product innovation and creating opportunities in mind, the two types of product innovation, exploitative and exploratory innovation, have attracted the attention of numerous researchers (Wang et al., 2019) and, in due course, innovation ambidexterity and ambidextrous innovation became two academic-valuable streams (Wang et al., 2019). Innovation ambidexterity combines exploitative and exploratory innovation in varied formulations and refers to a firm’s ability to pursue both types of innovation (He and Wong, 2004; Dunlap et al., 2016). In contrast, ambidextrous innovation indicates the activities an organization undertakes to develop different innovation (Khan et al., 2019). Innovation ambidexterity, regarded as dynamic capability, can improve firms’ innovation with effectiveness and efficiency, which, in turn, contributes to a firm’s competitive advantage according to Wang et al. (2019). It is also difficult for competitors to imitate firms’ innovation processes that are characterized by innovation ambidexterity (Zhang et al., 2016). Previous studies suggest that innovation ambidexterity can be improved not only through the component of the new knowledge and resources but also through new combinations of existing knowledge and resources (Wang et al., 2019). Three dynamic pathways, namely, exploitative and exploratory innovation simultaneously increasing, exploitative rather than exploratory innovation increasing, and exploratory rather than exploitative innovation increasing, are used to enhance innovation ambidexterity, putting the new component and the new combination together (He and Wong, 2004; Dunlap et al., 2016; Khan et al., 2019). Therefore, this study follows Wang et al. (2019) and utilizes innovation ambidexterity as the measure of firm-level outcomes, although both streams are meaningful and have numerous follow-up studies.

### Upper Echelons Theory Combined With the Entrepreneurial Cognition Approach

Hambrick and Mason’s seminal study (Hambrick and Mason, 1984) caused considerable scholarly attention to be focused on the characteristics of top managers as pivotal factors influencing a company’s strategic decisions. Observable characteristics, psychological features, and interactions with others were three dominant aspects of chief executive officers (CEOs) discussed by researchers (Bromiley and Rau, 2016). Observable characteristics focused on the demographics of CEOs such as experience (Crossland et al., 2014), educational qualifications (Lewis et al., 2014), origins (Zhang and Rajagopalan, 2010), succession (Quigley and Hambrick, 2012), and gender (Smith et al., 2013). The facet of psychological features assessed the foundational mental and emotional qualities of CEOs, such as narcissism (Chatterjee and Hambrick, 2007), hubris (Li and Tang, 2010), and overconfidence (Billett and Qian, 2008), also attracted considerable scholarly attention. Finally, in the third aspect, power associations (Galema et al., 2012) and social ties (Crossland and Hambrick, 2011) of CEOs have also attracted substantial scholarly attention.

Following the psychological characteristics stream, the cognitive character of an entrepreneur inevitably influences firm-level outcomes because of strategic decision-making (Hambrick and Mason, 1984; Hambrick, 2007; Bromiley and Rau, 2016). In other words, entrepreneurs are assumed to be “information workers” who spend their time processing information about issues and opportunities. Therefore, mechanisms linking an entrepreneur’s cognition with strategic decision-making, such as an entrepreneur’s risk preference, have been investigated on the basis of behavioral agency theory (Wiseman and Gomez-Mejia, 1998; Pepper and Gore, 2015). In general, consistent with the theory of behavioral agency, the way in which an entrepreneur thinks and behaves depends on entrepreneurial cognition, which is defined as the knowledge scheme used by an entrepreneur to make assessments, judgments, or decisions involving opportunity evaluation, venture creation, and growth (Mitchell et al., 2002).

The above literatures divulge two main issues that have been addressed by researches on entrepreneurial cognition: cognition structure and cognition style (Mitchell et al., 2002; Brymer et al., 2011; Zamberi et al., 2014; Sassetti et al., 2018). Cognition structure, also called knowledge structure (Walsh, 1995), mental model (Daft and Weick, 1984; Fahey and Narayanan, 1989), or cognitive map (Dutton et al., 1983; Dutton and Jackson, 1987), represents the content and organization of knowledge (Mitchell et al., 2002). A cognition structure results from cumulative experiences and learning an entrepreneur has encountered in a specific domain (Gaglio, 1997). The cognition structure determines how an entrepreneur responds to new information (Gaglio and Katz, 2001). Cognition style is defined as “the consistent individual differences in preferred ways of organizing and processing information and experience” (Messick, 1976). The extant literature recognizes two camps of cognition style: unitary and dual-process. The unitary view indicates entrepreneurial decision-making through reliance on one single psychological process; the dual-process view highlights the use of two distinct but complementary cognitive systems by an entrepreneur to process information (Baldacchino et al., 2015). Generally, cognition style determines how an entrepreneur processes information in the course of strategic decision-making. However, although cognitive style is “consistent,” an entrepreneur must usually select a specific information processing system according to situations (Hayes and Allinson, 1994, 1998; Kozhevnikov, 2007).

LOC and ROC, as components of ACC and MCC, play critical roles in influencing entrepreneurial cognition through both cognition structure and cognition style. As regards the cognition structure, LOC can help to scan and process the experience about the failure, which is used to shape entrepreneurs’ knowledge structure. Different from the role of LOC, ROC can help to eliminate the negative emotion’s effect on the experience of learning from business failure, although it does not shape entrepreneurs’ knowledge structure directly. In addition, LOC and ROC elicit entrepreneurs’ different types of information processing systems as regards cognition style. LOC leads entrepreneurs to opt for analytical information processing system generally, while ROC usually causes an entrepreneur to choose intuitive information processing system (Burns and D’Zurilla, 1999). However, the knowledge is still dearth of the effect of coping combinations, despite the aforementioned relationships between LOC/ROC and entrepreneurial cognition. Hence, the roles of ACC and MCC are investigated in this study.

### Hypothesis Development

#### The Relationship Between ACC and Innovation Ambidexterity

The logic underlying the relationship between ACC and innovation ambidexterity is cognition structure shaping. According to Walsh (1995), formation mechanisms still exist even though the cognition structure of an entrepreneur is inherent. Specifically, an entrepreneur’s cognition is usually shaped through top-down (theory-driven) and bottom-up (data-driven) approaches. The top-down approach generates the cognitive structure of an entrepreneur from experiences, while the information itself shapes the entrepreneur’s cognitive structure with the bottom-up approach (Walsh, 1995). Given the limited attention of an entrepreneur, the top-down approach is dominant in most situations. The cognition structure of an entrepreneur, with the top-down approach, is usually shaped by the experience of learning from business failure. After the cognition structure was revised, two logics underline the improvement of innovation ambidexterity to pursue the new component and the new combination. The first logic is that entrepreneurs can acquire more potentiality to create opportunities, which in turn enhances both exploitative and exploratory innovation through adding components (Zhang et al., 2016; Wang et al., 2019). The second logic is that entrepreneurs can make more appropriate decisions to allocate innovation resources effectively, which, in turn, enhances both exploitative and exploratory innovation through the new combinations. Therefore, to uncover the relationship between ACC and innovation ambidexterity, the underlying logic of ACC on considered, so as to determine entrepreneurs’ strategic decision about firms’ opportunity creation and innovation resources allocation.

LOC and ROC continue to play critical roles, as ACC is the alignment shifting between LOC and ROC with a balanced relationship. Because the negative emotions and positive experience learning of business failure exist simultaneously, an entrepreneur utilizes LOC to learn from business failure and utilizes ROC to recover from the grief triggered by business failure (McGrath, 1999; Cope, 2011; Ucbasaran et al., 2013). Specifically, LOC, defined as “working through and processing aspects of a loss to break the emotional bonds to the object lost” (Shepherd et al., 2011, p. 1234), provides an entrepreneur with knowledge about business failure with which to revise “their belief systems.” In contrast, ROC, described as the suppression of negative feelings from loss through the avoidance of thought and through the focusing of attention to secondary sources of stress that arise from the losses (Shepherd, 2003), is not directly related to learning from business failure, yet it can help entrepreneurs restrain negative emotions which hinder entrepreneurs’ learning from the business failure.

The cognition structure shaping mainly underlies the relationship between ACC and innovation ambidexterity, although the above individual roles are still working. On the one hand, as a balanced combination between LOC and ROC, ACC provides a new effective coping for an entrepreneur to learn well with LOC and simultaneously and equally to recover well with ROC from the business failure. ACC, according to the definition, is conceived as an equal ambidextrous combination of LOC and ROC. The essence of ambidexterity is “to be able to play equally well with either hand” (Wang et al., 2019); the use of ACC thus indicates that the relationship between LOC and ROC is equal and in contradiction. For instance, Shepherd et al. (2011) indicated that switching well between LOC and ROC provided gains from reducing entrepreneurs’ negative emotions and increasing their information-processing capability. ACC, on the other hand, is an easy and kind rule-of-thumb for entrepreneurs to use. ACC provides an entrepreneur with an equally proportional combination between LOC and ROC to obtain the benefits and suppresses the negative emotions. The equal proportion makes it convenient for an entrepreneur to manipulate to learn from the business failure and recover from the grief triggered by business failure. Thereafter, the new component and the new combination to pursue both exploitative and exploratory innovation is increased with entrepreneurs’ shaped cognition structure by ACC, which in turn improves innovation ambidexterity (Zhang et al., 2016; Danneels and Braver, 2018). Accordingly, entrepreneurs can make more appropriate strategic choices to improve innovation ambidexterity, with the reshaped cognition structure through knowledge learned from business failure. Thus,

H1: Innovation ambidexterity increases in congruence with the alignment coping combination.

#### The Relationship Between MCC and Innovation Ambidexterity

The underlying logic of the relationship between MCC and innovation ambidexterity is information processing system eliciting. Entrepreneurs with different cognitive styles prefer different types of information processing systems, according to the dual-process view in cognition style research. To make the statement in detail, cognitive style and information processing system are more specifically elaborated. Cognitive style is defined by Messick (1976) as a rigorous concept in the early stage. Later on, the notion of cognitive style was classified into two categories: analytic and intuitive cognitive style (Ornstein, 1977). The followed research investigations indicated that both cognitive styles denoted separate modes of information processing served by distinct cognitive systems (Epstein, 2003). With regard to information processing, cognitive-experiential self-theory postulates two fundamentally parallel and interactive information processing systems: analytical and intuitive information processing (Epstein, 2003). Studies indicate that an entrepreneur with an analytic cognitive style prefers analytical information processing, linking the two streams of literature together (Sadler-Smith and Badger, 1998; Kickul et al., 2009), whereas an entrepreneur with an intuitive cognitive style evinces intuitive information processing (Sadler-Smith and Badger, 1998; Kickul et al., 2009).

Although cognitive style is consistent, evidences suggest that an entrepreneur does not always process the information in the same manner. An entrepreneur usually takes modifications according to situations (Hayes and Allinson, 1994, 1998; Kozhevnikov, 2007). In addition, although both analytical and intuitive information processing systems are usually integrated through seamless interaction, they sometimes struggle against each other. One of the two systems could be in a relative status of dominance. Consequently, the argument is which one of the two systems could be in a relative dominance status. According to Epstein (2003), the extent of the predominance is determined by numerous parameters such as an individual’s preference for each, and the person’s customary way of responding to situations. Among these parameters, coping is a key point. Burns and D’Zurilla (1999) indicated that LOC led entrepreneurs to opt for the analytical information processing system generally, while ROC usually caused an entrepreneur to choose the intuitive information processing system. In other words, coping has a significant correlation with an entrepreneur’s information processing system, specifically, the analytical information processing system is run with LOC and intuitive information processing system is run with ROC.

Either the analytic information processing system or the intuitive information processing system, with the misalignment between LOC and ROC, could dominate entrepreneurs’ cognitive style. Specifically, the analytic information processing system increasingly comes to a relative dominance status with MCC as LOC increases and ROC decreases, whereas the intuitive information processing system gradually dominates entrepreneurs’ cognitive style with MCC as LOC decreases and ROC increases. Furthermore, an entrepreneur’s strategic decisions pertaining to the allocation of innovation resources could be influenced by the dominance status of information processing system, which, in turn, improves either exploitative or exploratory innovation through the new component and combination. According, exploitative rather than exploratory innovation increases with MCC as LOC increases and ROC decreases, or exploratory rather than exploitative innovation increases with MCC as ROC increases and LOC decreases. This principle underlies the improvement of innovation ambidexterity (He and Wong, 2004; Dunlap et al., 2016; Khan et al., 2019).

The roles of two types of MCC on innovation ambidexterity, following the above theoretical logic, could be revealed in detail. Entrepreneurs tend to opt for the analytical information processing system generally with the MCC as LOC increases and ROC decreases, which leads to more innovation resources to exploitative innovation. Extant research explains that exploitative innovation is the extent to which a firm recombines existing knowledge to pursue innovation for the existing needs of customers (Jansen et al., 2006). In line with this definition, exploitative innovation is associated with standardization, efficiency, and incremental innovation. Accordingly, information convergence (Smith and Tushman, 2005) is essential for it to occur. Since an entrepreneur who opts for an analytical information processing system follows a structured approach to solve problems, such an entrepreneur is more likely to engage in exploitative innovation and allocate more innovation resources to exploitative innovation (De Visser and Faems, 2015), which in turn contributes more to the firm’s exploitative innovation capabilities. Therefore, the firm’s innovation ambidexterity would be enhanced through the pathway of increasing exploitative rather than exploratory innovation. Put differently, innovation ambidexterity would increase in congruence with MCC as LOC increased and ROC decreased.

In contrast, with regard to the MCC as LOC decreases and ROC increases, entrepreneurs tend to choose the intuitive information processing system, which leads to more innovation resources to exploratory innovation. Exploratory innovation is the extent to which a firm recombines new knowledge to pursue innovation for emerging customers or markets (Jansen et al., 2006), which is associated with creativity, improvisation, and radical innovation. Accordingly, information divergence (Smith and Tushman, 2005) is essential to its occurrence. An entrepreneur who chooses intuitive information processing system would prefer an open-minded approach to solve problem and would thus be more likely to engage in exploratory innovation (De Visser and Faems, 2015). Accordingly, an entrepreneur who follows an intuitive information processing system would tend to allocate more resources to exploratory innovation, to improve the firm’s exploratory innovation capabilities, and would ultimately improve the firm’s innovation ambidexterity through the pathway of increasing exploratory rather than exploitative innovation. Simply put, innovation ambidexterity would increase in congruence with MCC as ROC increased and LOC decreased. Thus,

H2: Innovation ambidexterity increases in congruence with misalignment combination coping.
H2a: Innovation ambidexterity increases as loss orientation coping increases simultaneously with restoration orientation coping decreasing.
H2b: Innovation ambidexterity increases as restoration orientation coping increases simultaneously with loss orientation coping decreasing.

## Methods and Measure

### Sample

The data for this study were collected through two serial-wave surveys conducted in China’s BER. Located in northern and northeastern China, BER comprises five provinces and municipalities (i.e., Beijing, Tianjin, Hebei, Liaoning, and Shandong). This region accounts for 18.2% of China’s GDP. Due to an abundance of technology, human capital, investment, as well as the policy of the area, BER is one of the most active entrepreneurship zones in China (Lin and Wang, 2019). Therefore, BER is an ideal area for the study of entrepreneurship, and it was appropriate to conduct the current investigation in this region.

The questionnaires used to collect data were designed in advance, after which a professional research company was hired to administer the survey. Before the data collection commenced, both the research team and the professional research company communicated intensively regarding the sample selection and data collection plan. A well-trained investigative team was then given the responsibility for data collection from the industrial clusters of the five provinces and municipalities. Face-to-face interviews were conducted to guarantee response quality (e.g., answer accuracy, data completeness, etc.), with investigators charged with providing the responding entrepreneurs with accurate explanations apropos questions and items.

The first wave of the survey was conducted between September 2017 and February 2018, and 988 responses were gathered in this round. Of the 988 samples, 677 respondent entrepreneurs recorded no business failure experience and 311 entrepreneurs reported experiencing business failure with their former businesses being closed down or sold-out. Therefore, only 311 entrepreneurs met the failure experience criterion and were considered for the follow-up survey. With business failure defined as a former business closed down or sold-out in accordance with Eggers and Lin (2015), a follow-up investigation was conducted between September 2018 and February 2019 with the 311 entrepreneurs who met the failure experience criterion of the study, but only 135 entrepreneurs from the original data set could be reached. Of the 135 respondents, the data entered by 29 entrepreneurs were found to be deficient. Therefore, 106 valid samples were ultimately used to test the study’s hypotheses.

A total of 311 interviews were conducted in the first wave of the survey. With 176 entrepreneurs not having been reached, 135 interviews were conducted in the second wave of the survey. Therefore, non-response bias was tested by comparing the 176 respondents participating only in the first wave of the survey with the 135 respondents participating in the two waves of the survey in terms of asset and number of employees (Armstrong and Overton, 1977). No significant differences were found. The sample distribution is presented in Table 1. Most of the entrepreneurs re-ventured when they were aged between 41 and 50; a majority of them had graduated from high school or a specialized secondary school followed by junior college. Most of the ventured firms employed less than 20 persons, followed by companies that engaged 21–40 personnel, In addition, the total assets of most of the ventured firms were less than 1,000 thousand yuan, followed by firms with 1,001–2,000 thousand yuan in assets.

**TABLE 1:** Sample description.

**Age (%)**	
20–30	1.887
31–40	23.585
41–50	45.283
51–60	29.245
61 and above	0.000
**Education (%)**	
Junior high school	7.547
High school or equal	51.887
Junior college	31.132
Bachelor’s degree	9.434
Postgraduate and above	0.000
**Employee (%)**	
1–20 persons	41.509
21–40 persons	16.038
41–60 persons	13.208
61–80 persons	9.434
81–100 persons	7.547
101 and above persons	12.264
**Asset (%)**	
0–1000 thousand yuan	34.906
1001–2000 thousand yuan	30.189
2001–3000 thousand yuan	12.264
3001–4000 thousand yuan	9.434
4001 and above thousand yuan	13.208

### Measure

*Innovation ambidexterity* refers to a firm’s capability of pursuing exploitative and exploratory innovation in the manner of a trade-off (Jansen et al., 2009; Zhang et al., 2016; Wang et al., 2019). While the conceptualization only provides the basic idea of operationalization, there is currently no widely accepted measurement for innovation ambidexterity. This study measured the concept in two stages in accordance with Wang et al. (2019).

The first step involved measuring exploitative innovation and exploratory innovation through items adapted from Jansen et al. (2006) on the understanding that the two concepts underpinned innovation ambidexterity. Keeping the definition of exploitative innovation in mind, three items were adapted for the measurement of exploitative innovation: (a) my firm regularly implements small adaptations to existing products and services; (b) my firm improves the efficiency of products and services; (c) my firm expands services for existing customers. Exploratory innovation was measured through the following three items: (a) my firm accepts demands that go beyond existing products and services; (b) my firm invents new products and services; (3) my firm frequently utilizes new opportunities in new markets. The entrepreneurs were required to respond on a 5-point Likert scale (1 = *completely disagree*, and 5 = *completely agree*).

In the second step, the index was computed to measure innovation ambidexterity. Three approaches were used to combine the measurement for innovation ambidexterity: the additive (Jansen et al., 2009; Wang et al., 2019), the multiplicative (Cao et al., 2009; Jansen et al., 2012), and the subtractive (He and Wong, 2004; Cao et al., 2009). The additive approach was adopted for the purpose of this study in accordance with Jansen et al. (2009). In this study, innovation ambidexterity reflects the aggregate of the summarized magnitudes of exploitative innovation and exploratory innovation. Specifically, the mean of the three items was computed to attain the respective scores for exploitative and exploratory innovation. Then, the mean value of exploitative and exploratory innovation was computed as the measurement index of innovation ambidexterity.

To guarantee reliability, Cronbach’s α and the composite reliability of exploitative and exploratory innovation were computed. Cronbach’s alpha was 0.807, 0.608, and the composite reliability was 0.810 for exploitative innovation and 0.644 for exploratory innovation, indicating an acceptable level of reliability (Fornell and Larcker, 1981; Kim et al., 2012; Lam, 2012). In addition, the average variance extracted (AVE) was computed, and the values were 0.588 for exploitative innovation and 0.415 for exploratory innovation. The value of 0.588 indicates that the convergent validity of exploitative innovation was acceptable. However, even though the 0.415 value for exploratory innovation was below the recommended level of 0.5, it was above 0.4, which is higher than the acceptable level for Kim et al. (2012).

#### Alignment Coping Combination and Misalignment Coping Combination

Coping refers to cognitive and behavioral efforts made by people to manage external and internal demands that are appraised as stressful (Lazarus and Folkman, 1984). Coping is traditionally classified as problem-focused and emotion-focused coping (Biggs et al., 2017). LOC indicates working through some aspect of loss experience and ROC indicates avoiding feelings of loss and turning toward secondary sources of stress. These concepts were utilized in the present study to compute the ACC and MCC using polynomial regression with response surface analysis (Edwards and Parry, 1993; Shepherd, 2003; Shanock et al., 2010; Shepherd et al., 2011).

Three items were adapted to measure LOC: (a) In my mind, I often go over the events leading up to the failure; (b) I confront my thoughts about the failure; (c) I work through negative emotions generated in me by the failure. The items were adopted from the “self” dimension of the LOC scale developed by Shepherd et al. (2011). Similarly, three items were adapted from the “avoidance” dimension for the measurement of ROC from the scale developed by Shepherd et al. (2011): (a) I deliberately distract myself from thinking about the failure; (b) I seek people who talk about topics unrelated to the failure; (c) I keep my mind active so it does not focus on the failure.

The responses to the items were on 5-point Likert scales ranging from *completely disagree* to *completely agree*. The means of the three items from LOC and the three items from ROC were then computed to attain the final scale scores for each. For LOC and ROC, Cronbach’s α was 0.896 and 0.784, composite reliability was 0.899 and 0.809, and the AVE was 0.749 and 0.599, respectively. The results indicated that reliability and convergent validity were both acceptable; all the squared roots of the AVE were greater than all the corresponding correlation coefficients in Table 2, evidencing an acceptable discriminant validity.

**TABLE 2:** Means, standard deviations, and correlations.

**Variables**	**Mean**	***SD***	**IA**	**LOC**	**ROC**	**Age**	**Education**	**Employee**
Innovation ambidexterity (IA)	4.135	0.751	–					
Loss orientation coping (LOC)	2.840	1.062	0.130	0.865				
Restoration orientation coping (ROC)	2.660	0.884	0.142	0.393**	0.774			
Age	3.019	0.780	–0.086	–0.115	–0.179	–		
Education	3.425	0.768	–0.023	0.010	0.224*	−0.379**	–	
Employee	2.623	1.791	–0.186	−0.209*	–0.118	0.257**	0.000	–
Asset	2.102	0.515	–0.044	−0.396**	–0.064	0.247*	0.074	0.577**

The square root of AVE for each construct is on the diagonal. SD is the abbreviation of standard deviations. Significance level: *p < 0.05; **p < 0.01 (two-tail tests, sample size = 106).

#### Control Variables

Two entrepreneur level variables (i.e., age and education) were included in the analysis. These variables were also integrated into previous studies on business and project failure (Shepherd et al., 2011; Eggers and Lin, 2015; Lin and Wang, 2019). *Age* was measured in five categories (1 for 20–30, 2 for 31–40, 3 for 41–50, 4 for 51–60, and 5 for 61 and above); *education* was measured in eight categories (1 for primary school, 2 for junior high school, 3 for high school/specialized secondary school, 4 for junior college, 5 for undergraduates, 6 for master’s, 7 for doctorate, and 8 for others). Two firm-level variables, employee and asset, were also incorporated. The employee variable was measured in six categories (1 for 1–20 persons, 2 for 21–40 persons, 3 for 41–60 persons, 4 for 61–80 persons, 5 for 81–100 persons, and 6 for 101 and above), while the asset variable was measured by the logarithm of the firm’s total asset value (Eggers and Lin, 2015; Lin and Wang, 2019).

### Common Method Variance

The data were collected using a questionnaire and, therefore, the common method variance was controlled and assessed as per Podsakoff et al. (2003). Firstly, the design procedure used in this study avoided CMV. The data for the independent variables were collected from the first wave survey and those for the dependent variable were collected from the second wave survey to avoid CMV by combining data from different time horizons. In addition, CMV was controlled through the methodological separation of measurement. Five Likert scales were employed to measure exploitative innovation, exploratory innovation, LOC, and ROC. Statistical indicators were used to measure the control variables. Even though the above design procedures were adopted, Harman’s single-factor analysis was also performed to assess CMV (Podsakoff et al., 2003; Wang et al., 2019). An un-rotated factor analysis revealed that the first factor explained 33.665% of the variance, which was lower than the 50% cut-off.

### Analytical Techniques

To provide more specific explanations about the interactions between two independent variables, polynomial regression with response surface analysis was instead utilized to study the alignment effect (Edwards and Parry, 1993; Edwards, 2002; Cafri et al., 2010; Shanock et al., 2010). The polynomial regression with response surface analysis was conducted in two steps. The first stage examined the effects of the combination (***X*** and ***Y***) on the dependent variable, adding the lower-order variables (***X*** and ***Y***), high-order variables (***X*^2^**, ***XY***, and ***Y*^2^**), and control variables. The regression equation was:

$$\;\,Z=b_{0}+b_{1}\,X+b_{2}\,Y+b_{3}\,X^{2}+b_{4}\,\mathrm{XY}+b_{5}\,Y^{2}$$

    +Σ⁢c⁢Ω+e

In the equation, ***Z*** referred to innovation ambidexterity; ***X*** was LOC and ***Y*** was ROC; and ***X*^2^**, ***Y*^2^**, and ***XY*** were their squared terms and their product, respectively, **Ω** indicated the vector of the control variable including age, education, employee, and asset. The values of ***X*** and ***Y*** were mean-centered to aid interpretation (Edwards, 2002).

The second phase computed the four surface coefficients and tested the effects of alignment and misalignment (ACC and MCC). The alignment coefficients were indicated by ***a*_1_** and ***a*_2_** and the misalignment coefficients were designated as ***a*_3_** and ***a*_4_**. ***a*_1_**, defined as ***b*_1_ + *b*_2_**, reflected the slope of the line of perfect alignment; and ***a*_2_**, defined as ***b*_3_ + *b*_4_ + *b*_5_**, was the curvature along the line of perfect alignment. A significant positive (negative) ***a*_1_** revealed that the dependent variable ***Z*** increases (decreases) when both ***X*** and ***Y*** increase; a significant positive (negative) ***a*_2_** indicated a convex (concave) surface effect on the dependent variable ***Z*** with the alignment between ***X*** and ***Y*** increasing. ***a*_3_**, defined as ***b*_1_** - ***b*_2_**, was the slope of the line of misalignment, indicating the direction of the discrepancy between ***X*** and ***Y***; ***a*_4_**, defined as ***b*_3_** - ***b*_4_ + *b*_5_**, was the curvature of the line of misalignment, indicating the degree of discrepancy between ***X*** and ***Y***. A positive (negative) ***a*_3_** suggested that when ***X* > *Y*** (***X* < *Y***) the value of the dependent variable is higher than when ***X* < *Y*** (***X* > *Y***); a significant positive (negative) ***a*_4_** meant a convex (concave) surface effect on the dependent variable ***Z*** with the misalignment between ***X*** and ***Y*** increasing.

## Results

### Descriptive Analysis

Table 2 reports the means, standard deviations, and correlations. The correlation analysis revealed that LOC and ROC were positively correlated with innovation ambidexterity. The correlation coefficients were all moderate, indicating no obvious collinearity between variables. Therefore, the data can be used in regression analyses.

### Polynomial Regression With Response Surface Analysis

SPSS 25 was used in this study to conduct polynomial regression with response surface analysis using the RSA package (Edwards, 2002). The results are presented in Table 3. With regard to the effect of ACC, ***a*_1_** was found to be significantly positive (*p* = 0.040), indicating that innovation ambidexterity increases when ACC increases. In addition, ***a*_2_** was also significantly positive (*p* = 0.016), highlighting the existence of a convex surface effect on innovation ambidexterity with increasing ACC. Therefore, H1 is partially supported.

**TABLE 3:** Polynomial regression with response surface analysis.

**Variables**	**Coefficients**	***SE***
Constant (b_0_)	4.118***	0.588
Age	–0.070	0.105
Education	–0.089	0.103
Employee	0.286*	0.186
Asset	–0.108	0.050
Loss orientation coping (LOC) (b_1_)	0.053	0.085
Restoration orientation coping (ROC) (b_2_)	0.159	0.096
LOC squared (b_3_)	0.186**	0.063
LOC × ROC (b_4_)	–0.038	0.085
ROC squared (b_5_)	0.087	0.090
a_1_	0.212*	0.102
a_2_	0.235*	0.096
a_3_	–0.106	0.150
a_4_	0.311*	0.145

Dependent variable: innovation ambidexterity; significance level: *p < 0.05; **p < 0.01; ***p < 0.001 (two-tail tests, sample size = 106); a_1_ = b_1_ + b_2_, a_2_ = b_3_ + b_4_ + b_5_, a_3_ = b_1_ – b_2_, and a_4_ = b_3_ – b_4_ + b_5_, where b_1_ is the coefficient for LOC, b_2_ is the coefficient for ROC, b_3_ is the coefficient for LOC squared, b_4_ is the coefficient for LOC × ROC, b_5_ is the coefficient for LOC squared.

With reference to the effect of MCC, ***a*_4_** was significantly positive (p = 0.034), confirming a convex surface effect on innovation ambidexterity with MCC increasing. However, ***a*_3_** was not significant (*p* = 0.480), proving that the direction of the discrepancy between LOC and ROC is not significantly related to innovation ambidexterity. Therefore, H2 is supported.

**FIGURE 1:**
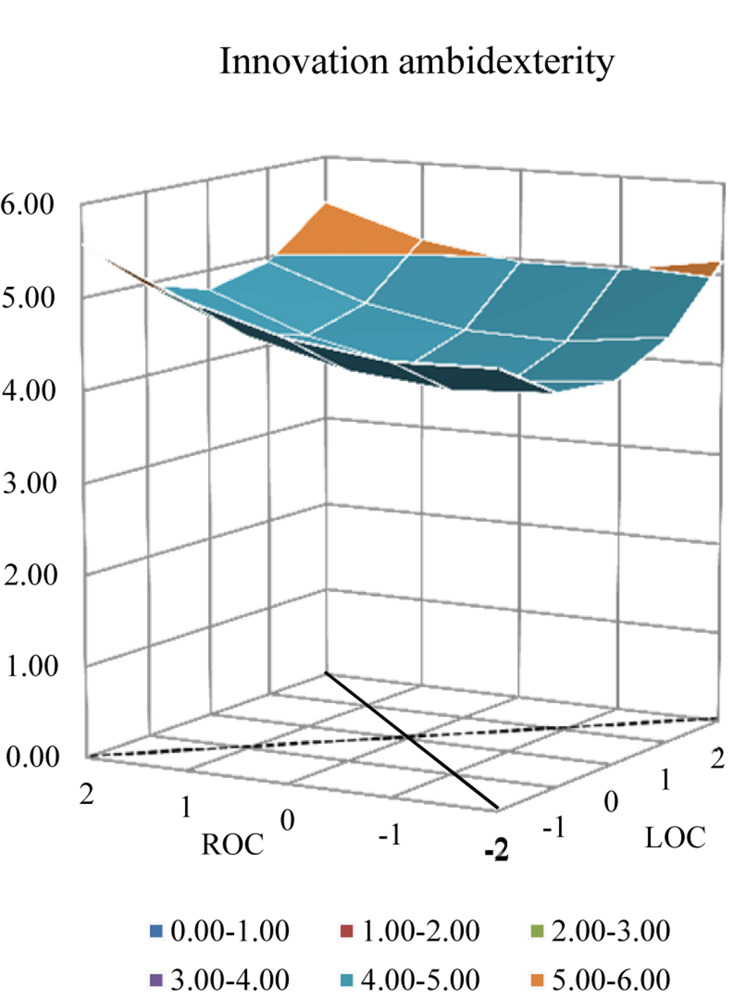
Response surface analysis of innovation ambidexterity.

**FIGURE 2:**
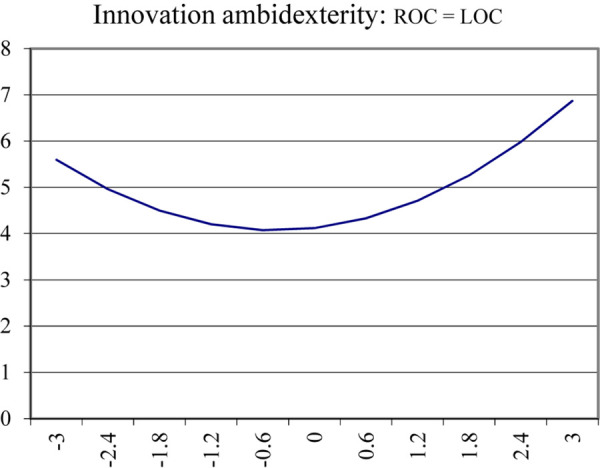
Innovation ambidexterity along the alignment.

The quadratic terms of response surface analysis revealed a complex relationship between ACC and innovation ambidexterity. Figure 1 demonstrates and details the complex pattern. Along the line of ACC, the higher value of innovation ambidexterity on the surface is at the corner where ACC is relatively high and low, and the lowest value is in the middle. The graph in Figure 2 shows an upward curving relationship between ACC and innovation ambidexterity. The result pertaining to high-level ACC is why H1 is partly supported.

**FIGURE 3:**
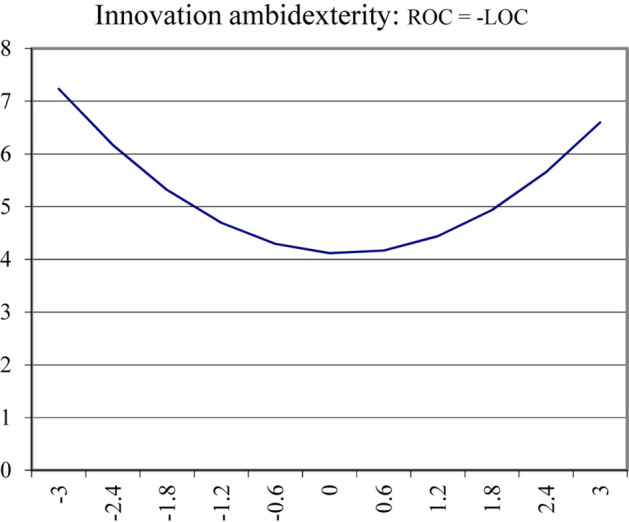
Innovation ambidexterity along the misalignment.

Along the line of MCC, the lowest value of innovation ambidexterity is in the middle; and innovation ambidexterity increases no matter ROC was higher than LOC or vice versa, that means innovation ambidexterity was higher than when ROC was equal with LOC. The graph in Figure 3 demonstrates that as LOC rises and comes closer to ROC, innovation ambidexterity decreases until it reaches the bottom (left side of the graph). However, when LOC continues to rise, innovation ambidexterity also sees an upward incline. Therefore, innovation ambidexterity increases with oscillations between LOC and ROC, which is why H2 is supported. Besides, although the graph shows an upward curving relationship between MCC and innovation ambidexterity, the discrepancy in innovation ambidexterity is not significantly different between the right and the left direction.

### *Post-hoc* Analysis

**FIGURE 4:**
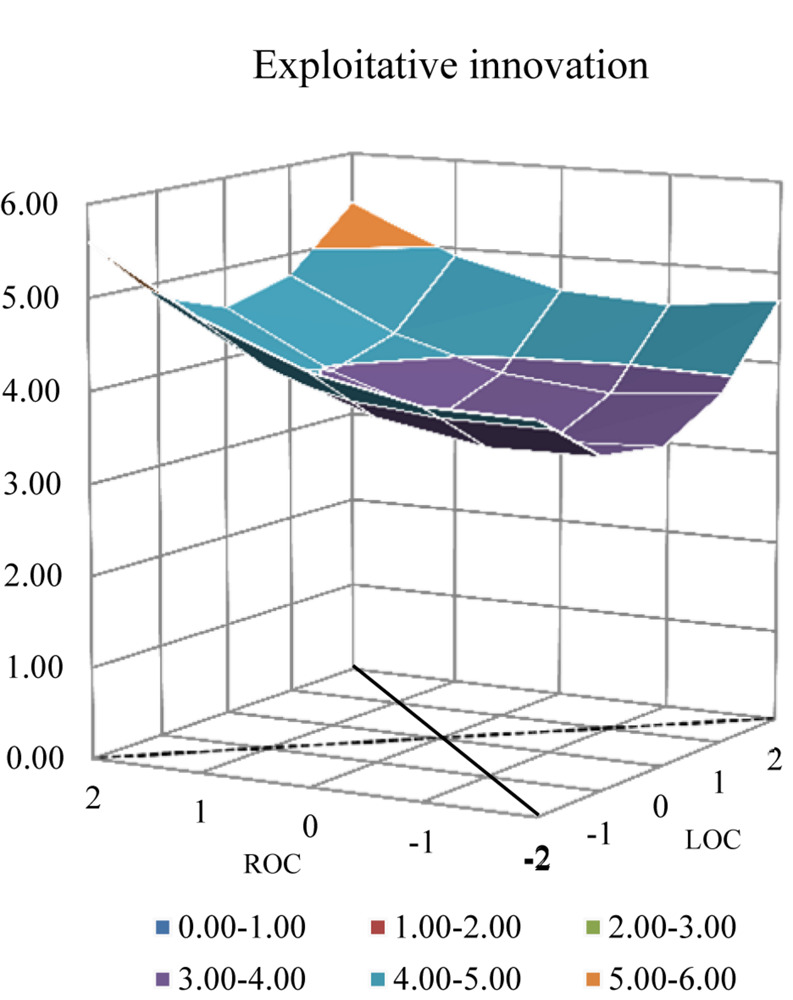
Response surface analysis of exploitative innovation.

**FIGURE 5:**
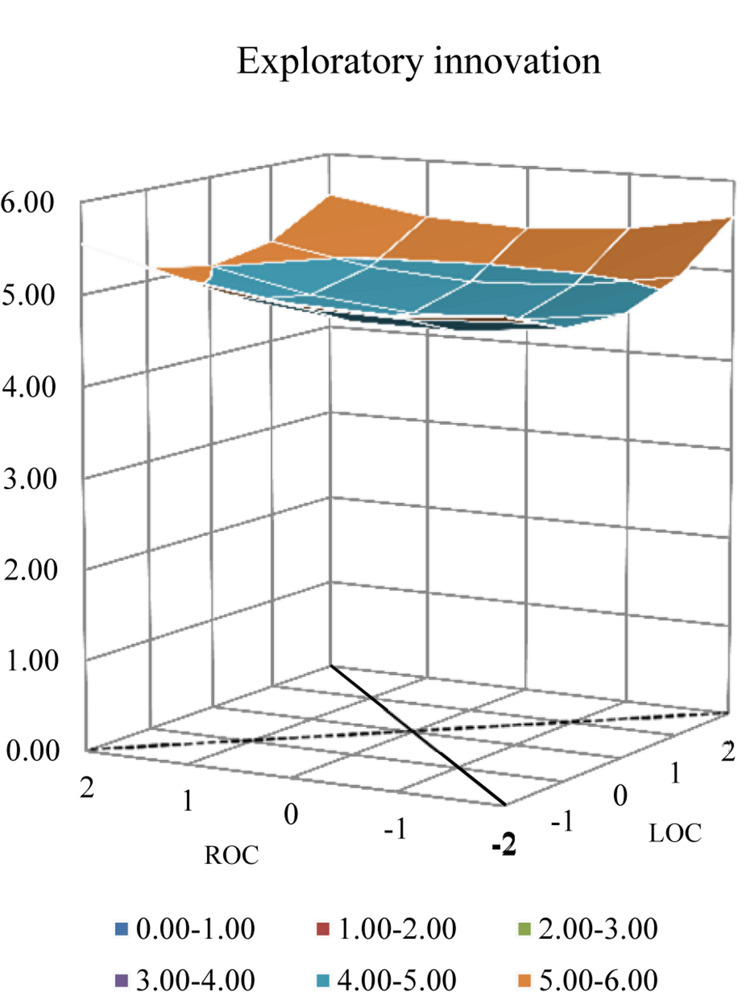
Response surface analysis of exploratory innovation.

Polynomial regression with response surface analysis was conducted respectively on exploitative and exploratory innovation. Table 4 exhibits the regression results and the response surface analysis graphs are displayed in Figures 4, 5. According to the regression analysis on exploitative innovation, both ***a*_1_** (*p* = 0.015) and ***a*_2_** (*p* = 0.025) were found to be significantly positive, along with ***a*_4_** (*p* = 0.032). In addition, the shape along the line of both ACC and MCC in Figure 4 was observed to be similar to the shape seen in Figure 1. Compared with innovation ambidexterity, these results indicate the similar pattern of the relationship between ACC (MCC) and exploitative innovation, except for the deeper “bowl” noted in Figure 4. In terms of exploratory innovation, ***a*_2_** (*p* = 0.056) and ***a*_4_** (*p* = 0.079) were marginally significant with positive parameters; however, ***a*_1_** was not significant. Further, although the shape of the pattern in Figure 5 was found to be similar to the form seen in Figure 1, the “bowl” in Figure 5 was much shallower, indicating a weak connection between ACC (MCC) on exploratory innovation.

**TABLE 4:** Polynomial regression with response surface analysis.

**Variables**	**Exploitative innovation**		**Exploratory innovation**	
	**Coefficients**	***SD***	**Coefficients**	***SD***
Constant (b_0_)	3.572***	0.732	4.664***	0.528
Age	–0.056	0.130	–0.083	0.094
Education	–0.074	0.128	–0.104	0.092
Employee	−0.121^†^	0.062	−0.095*	0.045
Asset	0.365	0.231	0.207	0.167
Loss orientation coping (LOC) (b_1_)	0.050	0.106	0.055	0.076
Restoration orientation coping (ROC) (b_2_)	0.276*	0.120	0.043	0.086
LOC squared (b_3_)	0.238**	0.079	0.134*	0.057
LOC × ROC (b_4_)	–0.045	0.106	–0.030	0.076
ROC squared (b_5_)	0.111	0.112	0.064	0.081
a_1_	0.326*	0.133	0.098	0.096
a_2_	0.304*	0.134	0.168^†^	0.087
a_3_	–0.226	0.183	0.012	0.131
a_4_	0.394*	0.181	0.228^†^	0.129

Dependent variable: exploitative innovation, exploratory innovation; significance level: ^†^p < 0.10; *p < 0.05; **p < 0.01; ***p < 0.001 (two-tail tests, sample size = 106); a_1_ = b_1_ + b_2_, a_2_ = b_3_ + b_4_ + b_5_, a_3_ = b_1_ – b_2_, and a_4_ = b_3_ – b_4_ + b_5_, where b_1_ is the coefficient for LOC, b_2_ is the coefficient for ROC, b_3_ is the coefficient for LOC squared, b_4_ is the coefficient for LOC × ROC, b_5_ is the coefficient for LOC squared.

**FIGURE 6:**
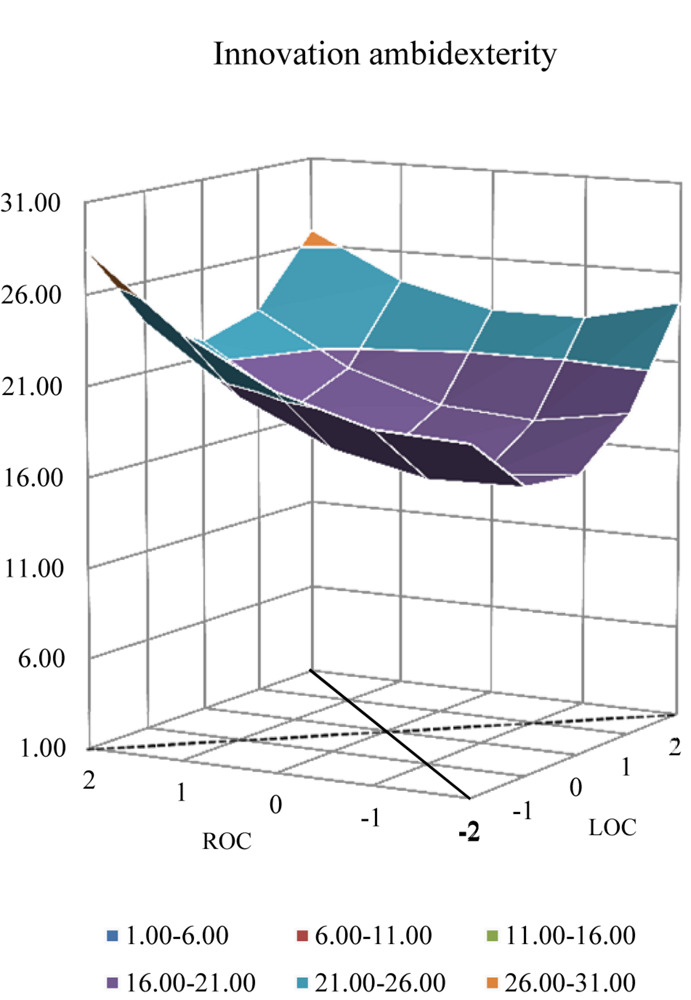
Response surface analysis of innovation ambidexterity (the multiplicative approach).

Two dimensions, namely, the combined dimension and the balanced dimension, were considered to measure innovation ambidexterity (He and Wong, 2004; Cao et al., 2009). The additive approach and the multiplicative approach were classified into the combined dimension, whereas the subtractive approach was classified into the balanced dimension. Polynomial regression with response surface analysis was also conducted on innovation ambidexterity according to the multiplicative approach, for that both additive and multiplicative approach belong to the same dimension. Table 5 presents the results, and the response surface analysis graph is displayed in Figure 6. Both ***a*_1_** (*p* = 0.021) and ***a*_2_** (*p* = 0.025) were found to be significantly positive, according to the regression analysis on innovation ambidexterity measured with the multiplicative approach, whereas ***a*_4_** (*p* = 0.060) was marginally significant. In addition, both the line of ACC and the line of MCC in Figure 6 had a similar shape to that observed in Figure 1. These results indicate the robustness of the pattern of the relationship between ACC (MCC) and innovation ambidexterity.

**TABLE 5:** Polynomial regression with response surface analysis (the multiplicative approach).

**Variables**	**Coefficients**	***SE***
Constant (b_0_)	16.658^∗∗∗^	4.224
Age	–0.375	0.752
Education	–0.525	0.739
Employee	−0.650^†^	0.357
Asset	1.883	1.334
Loss orientation coping (LOC) (b_1_)	0.355	0.610
Restoration orientation coping (ROC) (b_2_)	1.391^∗^	0.691
LOC squared (b_3_)	1.429^∗∗^	0.455
LOC × ROC (b_4_)	–0.382	0.611
ROC squared (b_5_)	0.626	0.645
a_1_	1.746^∗^	0.744
a_2_	1.673^∗^	0.761
a_3_	–1.036	1.070
a_4_	2.437^†^	1.280

**Dependent variable: innovation ambidexterity; significance level: ^†^p < 0.10; ^∗^p < 0.05; ^∗∗^p < 0.01; ^∗∗∗^p < 0.001 (two-tail tests, sample size = 106); a_1_ = b_1_ + b_2_, a_2_ = b_3_ + b_4_ + b_5_, a_3_ = b_1_ – b_2_, and a_4_ = b_3_ – b_4_ + b_5_, where b_1_ is the coefficient for LOC, b_2_ is the coefficient for ROC, b_3_ is the coefficient for LOC squared, b_4_ is the coefficient for LOC × ROC, b_5_ is the coefficient for LOC squared.**

## Discussion and Conclusion

### Complex Links Between Coping Combination and Innovation Ambidexterity

Despite emerging research on the effect of LOC and ROC at the individual level in situations of business failure (Shepherd, 2003; Shepherd et al., 2011; Shepherd and Patzelt, 2018), there still exists a dearth of knowledge with regard to the role of coping methods at the firm level. Thus, this study drew upon the upper echelons theory combined with the entrepreneurial cognition approach to examine how coping combinations (ACC and MCC) may be related to innovation ambidexterity in the event of a business failure.

Data were collected through two serial-wave surveys in the BER region of China and were analyzed using polynomial regression with response surface analysis technology. The analyses found empirical evidences to support the study’s hypotheses. The results of the study seem to align with the entrepreneurial cognition approach of the upper echelons theory (Hambrick and Mason, 1984; Randolph-Seng et al., 2015). According to this perspective, entrepreneurial cognition (thought structure and information processing system) influences an entrepreneur’s strategic decision. In support of the reshaping of an entrepreneur’s cognitive structure logic, the findings of the present study demonstrate that ACC is positively related to innovation ambidexterity when ACC is relatively high. In addition, the outcome of MCC being positively related to innovation ambidexterity is in support of the activating information processing system logic. In other words, coping mechanisms (ACC and MCC) influence the firm-level innovation ambidexterity through the underlying logic of entrepreneurial cognition.

Interestingly, the *post hoc* analysis reveals that the effects taken by coping combinations are different vis-à-vis exploitative and exploratory innovation. The “bowl” in Figure 4 is deeper, suggesting (Table 4) that both ACC and MCC are significantly related to exploitative innovation. In contrast, the “bowl” in Figure 5 is much shallower, indicating (Table 4) that both ACC and MCC are only marginally significantly associated with exploratory innovation. Therefore, it may be inferred that to some extent an entrepreneur’s attitude becomes relatively conservative after business failure and that an entrepreneur would prefer to allocate more resources to exploitative innovation activities.

In addition, although not hypothesized, the results demonstrate that innovation ambidexterity decreases with an increase in ACC when ACC is relatively low, which indicates that reverse effect occurs when ACC is relatively low. Perhaps the results are attribute to the downside of the switching, also known as the cognitive switching penalty in entrepreneurial cognition (Monsell, 2003): time and effort are wasted when an entrepreneur reorients coping strategies (Putnam et al., 2016). In this study, the reverse effect when ACC is relatively low may result from the fact that the costs of frequent switching between LOC and ROC are more than the benefits of the shifts. Therefore, the application of ACC should be carefully considered. The final effect of ACC, which is also a temporal ambidextrous oscillation, lies in its potential mechanisms concerning the positive and negative sides.

### Contributions

The study adds to the literature on coping strategies in business failure situations in several important ways. First, it contributes to the upper echelons theory with the entrepreneurial cognition approach (Dutton et al., 1983; Daft and Weick, 1984; Hambrick and Mason, 1984; Fahey and Narayanan, 1989; Walsh, 1995; Gaglio, 1997). Previous studies on the upper echelons theory addressed three aspects of the top managers: observable characteristics, psychological features, and interactions with others (Bromiley and Rau, 2016). The effects of narcissism (Chatterjee and Hambrick, 2007), hubris (Li and Tang, 2010), and overconfidence (Billett and Qian, 2008) on strategic decision-making have been examined as psychological features; however, most of the existing studies have been based on a narrow underlying logic of psychological characteristics. The present study focused on the effects of ACC and MCC on innovation ambidexterity through the entrepreneurial cognition approach and revealed the general underlying logic of psychological characteristics. This study, therefore, contributes to the upper echelons theory from the entrepreneurial cognition approach.

Second, this study supplements the fundamental theoretical logic of coping mechanisms at the organizational level. Although coping is quite commonly used to overcome the negative impact of business failure, research on this activity is still at an early stage (Shepherd, 2003; Shepherd et al., 2011; Shepherd and Patzelt, 2018). Previous studies have focused primarily on the effects of coping strategies at the individual level. They have investigated three categories of coping (loss orientation, restoration orientation, and oscillation orientation) and two focus classifications (problem-focused coping and emotion-focused coping) employed to overcome the negative effects of business failures (Shepherd, 2003; Singh et al., 2007; Shepherd et al., 2011). The present study integrated innovation ambidexterity into its research on coping and focused on the effects of ACC and MCC on innovation ambidexterity to investigate firm-level outcomes. The results of this study answered its research questions and revealed how ACC and MCC influence innovation ambidexterity. The findings of this investigation thus enhance scholarly understanding of the mechanism that underpins the effects of coping on innovation ambidexterity.

Third, this research introduced the effects of coping strategies described in business failure literature from the information processing perspective (Burns and D’Zurilla, 1999). Most previous studies have explored how coping functions on the basis of the functional analytic perspective (Shepherd, 2003; Singh et al., 2007; Shepherd et al., 2011). Only a few studies have investigated the role of coping strategies from the information processing perspective. The present study bridges this research gap by empirically applying the information processing perspective to the investigation of the effects of ACC and MCC on innovation ambidexterity. In congruence with Epstein (1990), the analytical information processing system is associated with the left-brain function of converging information and operates at the conscious level with an intentional, analytic, and primarily verbal nature. On the other hand, the intuitive information processing system is associated with the right-brain function of diverging information and operates in a manner that is automatic, preconscious, holistic, associative, and primarily non-verbal in character (Norris and Epstein, 2011). The findings of the present investigation enrich the theoretical logic of coping mechanism from both the analytical and intuitive information processing system perspectives.

In addition to the theoretical implications, this study also offers substantial practical implications. First, the obtained results provide entrepreneurs failed in previous business with more precise coping combinations through which they can learn from their business failures and simultaneously recover from the grief triggered by failed ventures. Second, the outcomes of this investigation can inspire entrepreneurs to control the critical role of entrepreneurial cognition, especially after a business failure. Indeed, the reshaping of the cognition structure and the selection of appropriate information processing system are both critical for an entrepreneur to benefit from ACC and MCC while minimizing the downsides of business failure. Third, the results imply the importance of exploratory innovation activities. Although the findings disclose that an entrepreneur’s attitude becomes relatively conservative after a business failure, the entrepreneur must allocate appropriate innovation resources to exploratory innovation activities, so that innovation ambidexterity may be more enhanced at the firm level.

### Limitations and Directions for Future Research

Like any other investigation, the present study must acknowledge certain limitations. First, the limited resources and difficulties in data collection resulted in a valid sample size of only 106 respondents even though the researchers tried their best to maximize the sample size. Neither the data of 61 and above years old nor the data of the education level higher than bachelor were acquired with the limited samples, although the research design included them. In addition, the measurement data of innovation ambidexterity should be better collected at the firm level, not just from the individual informant level. Future studies should invest more resources in the collection of data so that the causal relationships between coping combination and innovation ambidexterity can be comprehensively investigated. Second, some of the items of original scales were dropped to guarantee the reliability and the validity of the survey because of the contextual impact. To obtain more robust results, prospective studies should develop more appropriate scales to suit the Chinese context. Third, factors that play moderating roles may exist and create different effects of coping combinations on exploitative innovation and exploratory innovation. Forthcoming studies should explore these moderating factors between coping combination and innovation ambidexterity.

## Data Availability Statement

The datasets for this article are not publicly available because of the privacy of entrepreneurs. Requests to access the datasets should be directed to linsong1998@126.com.

## Ethics Statement

The studies involving human participants were reviewed and approved by the Academic Committee of business school in Central University of Finance and Economics on 01-April-2020. Written informed consent to participate in this study was provided by the patient/participants’ OR patient/participants legal guardian/next of kin.

## Author Contributions

JC participated in design, drafting of the first version, and revision of the article. FJ participated in design, revision of the article, and important intellectual input. SL participated in design, data collection, and important intellectual input. All authors contributed to the article and approved the submitted version.

## Conflict of Interest

The authors declare that the research was conducted in the absence of any commercial or financial relationships that could be construed as a potential conflict of interest.
